# Radiomics in Oncology: A 10-Year Bibliometric Analysis

**DOI:** 10.3389/fonc.2021.689802

**Published:** 2021-09-20

**Authors:** Haoran Ding, Chenzhou Wu, Nailin Liao, Qi Zhan, Weize Sun, Yingzhao Huang, Zhou Jiang, Yi Li

**Affiliations:** State Key Laboratory of Oral Diseases & National Clinical Research Center for Oral Diseases & Department of Head and Neck Oncology Surgery, West China Hospital of Stomatology, Sichuan University, Chengdu, China

**Keywords:** radiomics, oncology, bibliometric analysis, hotspots, trends

## Abstract

**Objectives:**

To date, radiomics has been applied in oncology for over a decade and has shown great progress. We used a bibliometric analysis to analyze the publications of radiomics in oncology to clearly illustrate the current situation and future trends and encourage more researchers to participate in radiomics research in oncology.

**Methods:**

Publications for radiomics in oncology were downloaded from the Web of Science Core Collection (WoSCC). WoSCC data were collected, and CiteSpace was used for a bibliometric analysis of countries, institutions, journals, authors, keywords, and references pertaining to this field. The state of research and areas of focus were analyzed through burst detection.

**Results:**

A total of 7,199 pieces of literature concerning radiomics in oncology were analyzed on CiteSpace. The number of publications has undergone rapid growth and continues to increase. The USA and Chinese Academy of Sciences are found to be the most prolific country and institution, respectively. In terms of journals and co-cited journals, *Scientific Reports* is ranked highest with respect to the number of publications, and *Radiology* is ranked highest among co-cited journals. Moreover, Jie Tian has published the most publications, and Phillipe Lambin is the most cited author. A paper published by Gillies et al. presents the highest citation counts. Artificial intelligence (AI), segmentation methods, and the use of radiomics for classification and diagnosis in oncology are major areas of focus in this field. Test-retest statistics, including reproducibility and statistical methods of radiomics research, the relation between genomics and radiomics, and applications of radiomics to sarcoma and intensity-modulated radiotherapy, are frontier areas of this field.

**Conclusion:**

To our knowledge, this is the first study to provide an overview of the literature related to radiomics in oncology and may inspire researchers from multiple disciplines to engage in radiomics-related research.

## Introduction

Unlike the natural intelligence displayed by humans and animals, artificial intelligence (AI) is intelligence demonstrated by machines. AI can be applied to develop systems possessing characteristics of human beings: the ability to learn, reasoning, sensing, and actioning. Initially, the development of automated interpretation of medical images was based on human decision models to perform high-level interpretations of images. At the time, logical rules were applied to AI machines. AI machines sought specific structures such as lines or circles for identification. Such systems have succeeded in other fields, such as in business and manufacturing. Next came the second generation of AI algorithms. Instead of focusing on certain symbols of images, such algorithms were designed to be more statistical. This kind of model of medical images may develop from healthy individuals, and its parameters are inferred from data. Such an algorithm can assist in helping radiologists identify lesions. The segmentation method serves as a classic example of this algorithm. Currently, the explosion of big data has ushered AI into a new era, and algorithms are called data-driven/model-free approaches, which involve automating knowledge discovery. This approach is now widely applied in medical research, and a popular application of this method is called radiomics (1).

Radiomics, first pioneered by Philippe Lambin (2), uses high-throughput data to extract certain features from medical images for personalized precision medicine development. With the development of AI, the field of radiomics has grown rapidly and been widely used in every phase of tumor treatment. Relying on quantitative data generated by medical imaging and the support of technology, radiomics offers a risk-free and efficient method for diagnosis (3, 4), classification (5), and prognosis prediction (6, 7) in oncology.

Data selection, medical imaging, feature extraction, exploratory analysis, and modelling are the five steps of radiomics (2, 8, 9). Applying standard imaging protocols to generate high-quality images from computed tomography (CT), positron-emission CT (PET-CT), magnetic resonance imaging (MRI), radiography or using high-quality photographs of lesions can enable radiomics reproducibility. Then, the volume of interest (VOI), which identifies lesions from images, is delineated by experienced radiologists or semiautomated or automated segmentation methods. The features extracted from VOIs are inputted to generate quantitative descriptions, which contain semantic and agnostic features. The value of extracted features is then analyzed, and only the features most contributing to the classifiers are retained for future modelling. In practice, this step can be supported by statistical approaches and AI, including a univariate analysis of variance, the least absolute shrinkage and selection operator, decision trees, neural networks, and support vector machines (9). The relationship among the algorithms is presented in **Supplementary Figure S1**.

Although radiomics can be applied in a large number of conditions, it is most well developed and widely used in oncology due to initial support received from the National Cancer Institute (9, 10) and Quantitative Imaging Biomarker Alliance (11). In 1973, some researchers intended to use texture features to classify images (12). In 1995, researchers started to use a convolutional neural network (CNN) to identify lung nodes, suggesting that it is possible to train computer algorithms to identify medical images (13). In late 2000, researchers attempted to identify the relationship between the imaging of tumors and their genomic types (14, 15). At the time, most studies were performed on relatively small datasets and lacked external examination, meaning that the established radiomic models were only based on small datasets from individual organizations and could not be validated by data from external organizations. With innovations made in the field of medical imaging, radiomics in oncology has rapidly progressed (16–19). In around 2012, radiomics was first proposed by Philippe Lambin; ever since, thousands of researchers have been encouraged to conduct radiomics-based research. In 2014, radiomics in oncology was used to examine CT imaging features for diagnosis and prediction (20). In 2016, researchers found for the first time that the radiomics signature could predict lymph node metastasis in patients with colorectal cancer (21). In 2018, researchers found that radiomics features could predict the treatment responses and prognosis of patients receiving immunotherapy (18). Some researchers have developed models for the automated identification of lesions from videos and images (3, 4). In addition, some researchers have found a correlation between radiomic features and tumor histology (22). At present, by extracting various features from medical images and translating these image features into high-throughput and quantitative data for analysis, radiomics can be used for the classification and differentiation of different lesions and subtypes of tumors (3–5) and for survival prediction (23) and prognosis prediction for patients undergoing radiation therapy (6, 24, 25). Even with common limitations, such as a lack of outside validations, the use of small datasets or the variabilities caused by medical imaging protocols, radiomics research has offered a significant opportunity for researchers to make clinical decisions from an entirely new perspective.

Since radiomics studies mostly rely on medical data, which are subject to approaches to data acquisition and analysis used, creating a gold standard for medical models remains a great challenge. When establishing a clinical model, the input and processing of radiomic features can drastically influence the model, as these features depend on the radiologists who record the clinical characteristics, on AI and on statistical methods. For example, inexperienced radiologists may fail to delineate a lesion entirely or miss significant clinical features, and different AI and statistical methods may create clinical diagnosis or prediction models with inconsistent accuracy. For these reasons, standardizing the procedures of radiomics studies and finding robust features with which to establish models are essential in achieving clinical goals of radiomics research.

Bibliometric analysis evaluates scientific activities in a certain field (26). A simple quantitative technique provided by citation analysis provides a means to estimate the impact of an article (27), such as the influence of bridging articles between themes or the influence of articles laying the foundation in certain fields. CiteSpace, developed by Chaomei Chen, is a Java-based application for detecting and visualizing possible trends and radical changes in scientific disciplines over time (28) and is a valuable tool for bibliometric analysis. The program can assist researchers in identifying influential and effective areas of research, trends, and prospects in certain fields. CiteSpace has been widely applied in many subjects for bibliometric analysis, such as neuroscience, oncology, and cardiovascular science (29–31).

In this article, we use data collected from the Web of Science Core Collection (WoSCC) and CiteSpace to analyze 7,199 publications related to radiomics in oncology and generated knowledge maps for the first time, to our knowledge. Since radiomics provides a new means for clinicians to examine entire tumors with rather minimally invasive methods, we sought to provide a more comprehensive understanding of the ever-changing field of radiomics. Furthermore, to encourage researchers from various disciplines to actively and creatively participate in practicing radiomics, we conducted our bibliometric analysis based on relevant literature in this field to outline the countries, authors, institutions, and journals that have made significant contributions to this field. In applying this method, we also identify areas of focus and future trends.

## Methods

### Data Acquisition and Search Strategy

Relevant literature was collected from the WoSCC. The following search terms were employed, the searching formula is also presented in **Supplementary Figure S2**.


(1) $$\begin{array}{l} \text{TS}=(\text{image}*\text{ OR picture}*\text{ OR photograph}*\text{ OR X−ray}*\text{OR CT OR MRI OR panorama }*\text{ OR}\\ \text{Computer Tomography OR Magnetic Resonance Imaging OR tomography OR PET CT}\\ \text{AND}\\ (\text{TS}=\;(\text{AI OR Artificial Intelligence OR deep learning OR machine learning OR computational}\\ \text{intellegen}*\text{ OR Convolutional Neural Network OR CNN})\text{ OR}(\text{TS}=\text{radiomic}*)\\ \text{AND}\\ \text{TS}=\;\left(\text{tumor}*\text{ OR cancer}*\text{ OR carcino}*\text{ OR onco}*\right) \end{array}$$

(2) $$\begin{array}{l} \left(\text{TS}=\text{radiomic}*\right)\\ \text{AND}\\ \text{TS}=\;\left(\text{tumor}*\text{ OR cancer}*\text{OR carcino}*\text{OR onco}*\right) \end{array}$$

The time interval was set to 2011 to 2020. Only articles and reviews were included, and no language restrictions were applied. The search and download process was carried out on March 14, 2021 to eliminate substantial errors caused by daily database updates. Given that data were directly downloaded from the database, ethical approval was not required.

### Data Analysis

CiteSpace V was used to remove duplicates and analyze the 7,199 unique records exported. Then, to visualize emerging trends and areas of focus in tumor radiomics research, CiteSpace was applied to generate knowledge mappings of countries, institutions, co-occurrences of keywords, references, authors and co-cited authors, and co-cited journals (28). With each year covered by a dataset assigned a different color, CiteSpace uses colorful node edges or crosses to discriminate between different research objects, including countries, institutions, words, references, etc. The size of rings on nodes indicates the number of publications for each node. Purple rings surrounding circles indicate the centrality of nodes. Moreover, “Burst detection” is a function provided by the software to detect current and prospective areas of focus. The detection objects of this function can include noun phrases, keywords included in abstracts, papers, and so on, revealing words, or papers undergoing citation bursts in a given period. When the time period is set to the present, this means that some keywords or papers are undergoing a citation burst, which may indicate further prospects for a given field. The impact factors (IFs) for all publications were documented based on the Journal Citation Report (2019).

**Figure 1:**
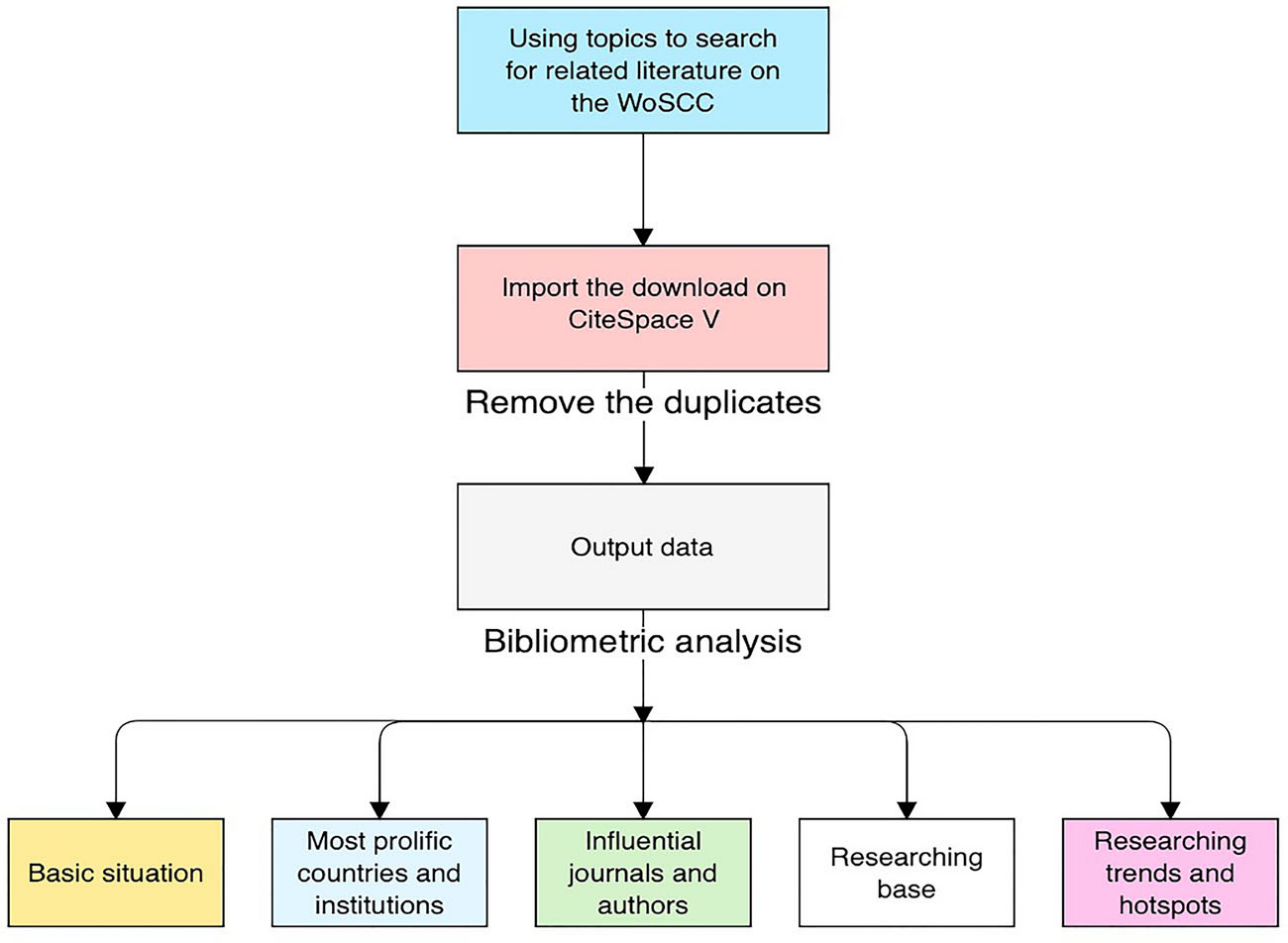
Workflows of this study.

Generally, the productivity of individuals, journals, and countries can be measured by the total number of papers, whereas the total counts of citations of authors, journals, or references measure the total impact. Defined as the maximum value of *h* such that the given author/journal has published at least *h* papers that have each been cited at least *h* times, the H index is used to characterize a researcher’s output in scientific research. The impact factor (IF), defined as a scientometric index, is also a measurement of journals and articles (32). The value is calculated as the average number of citations a publication receives in 2 or 5 years as indexed by the Web of Science. Furthermore, co-citation is defined as the number of times two documents are cited together (33); that is, when two publications or authors are cited at the same time, they may focus on the same theme of research, which may indicate their cooperation. A burst of an event refers to a surge in the frequency of a certain event, such as the emergence of a keyword or the citation of a specific article (34). These parameters allow us to identify productive institutions and their countries and outstanding individuals in the studied field. **Figure 1** illustrates the research steps of this study.

## Results

### State of Publication Output

**Figure 2:**
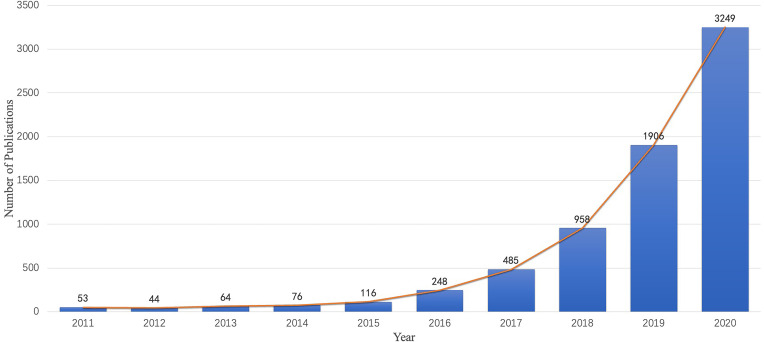
Chronological distribution of publications in radiomics for oncology from 2011 to 2020.

A total of 7,199 publications were examined in the present study and include 6,417 (89.1%) original articles and 782 (10.9%) reviews. **Figure 2** shows the chronological distribution of the publications for 2011 to 2020. With technological breakthroughs in AI, an increasing number of researchers have been attracted to radiomics in oncology. As depicted in the diagram, the number of articles and reviews grew steadily in the first 4 years. From 2015 to 2020, the annual number of publications grew exponentially and peaked in 2020.

### Active Countries and Institutions

The included publications were published in 82 countries and regions over the last decade. The top 10 contributors are presented in **Supplementary Table S1**, and the cooperative relationships among them are shown in **Supplementary Figure S3**. When counting the number of publications, the USA (2,280) ranks first, followed by China (2136), India (456), Germany (454), and England (401).

Overall, 715 institutions contributed to this field. **Supplementary Table S1** shows the 10 most productive institutions, and **Supplementary Figure S4** shows the cluster of institutions engaged in radiomics research in oncology by keyword. The most productive institution is the Chinese Academy of Sciences with 224 publications, followed by Sun Yat-Sen University (168), Fudan University (155), Harvard Medical School (145), and Stanford University (136). The three most prolific institutions are Chinese universities.

### Productive Journals

**Figure 3:**
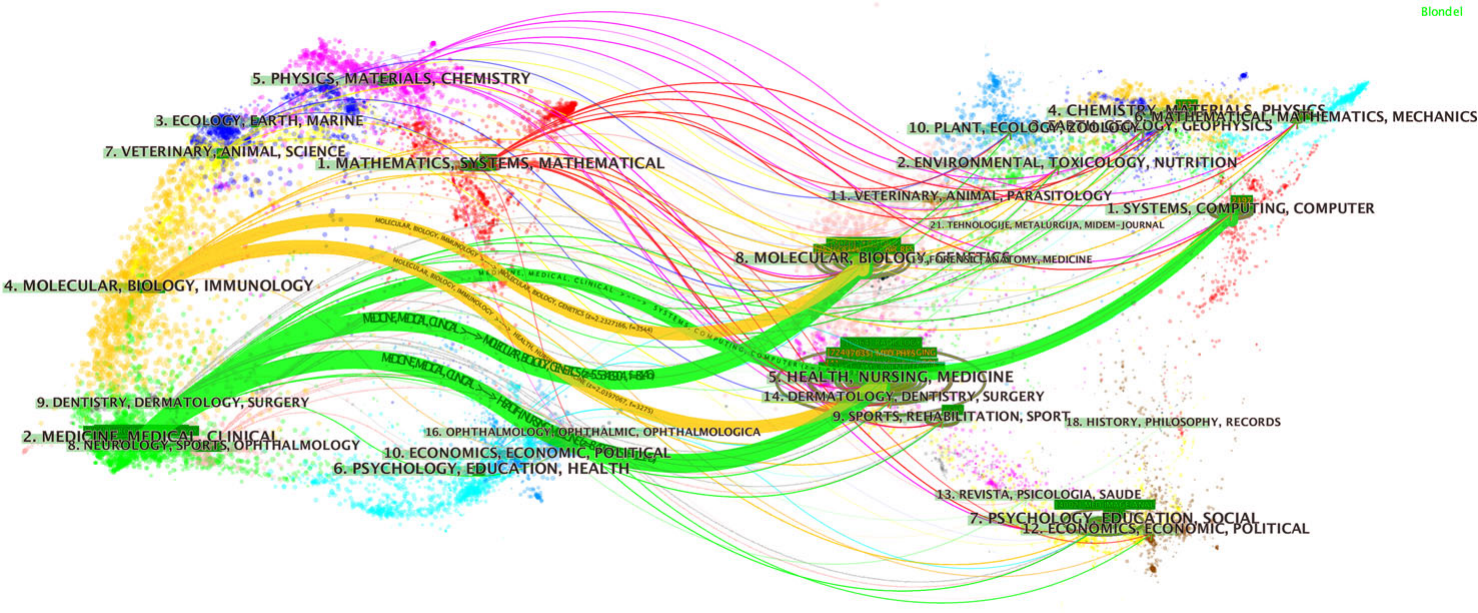
The dual map overlay of journals. This figure can be divided into two sections. Each dot represents one journal, and this knowledge map uses different colors to symbolize journals from different subjects. On the left, there are the citing journals of this field, and on the right, lays the cited journals in this field. The waves link to two sides means the publications on the journals of the left side may cite publications from the journals on the other side. For example, publications on journals in the field of medicine medical and clinical (labeled 2 on the left), may refer to the publications on the journal of systems, computing, and computer.

A total of 1,247 journals published articles or reviews in this field. We list the 10 most productive journals with their IFs in this field in **Supplementary Table S2** and provide an overdual map of citing and cited journals in **Figure 3**. According to statistics from the WoSCC, *Scientific Reports* published 253 publications over the last decade and thus ranks first. *Medical Physics* ranks second (244 publications), followed by *IEEE Access* (241 publications), *European Radiology* (213 publications), and *Frontiers in Oncology* (180 publications).

The 10 most co-cited journals are given in **Supplementary Table S2**. Among co-cited journals, *Radiology* was cited the most (3258 times), followed by *IEEE Transactions on Medical Imaging* (2800 times), *Scientific Reports* (2410 times), *PLoS One* (2407 times), and *Medical Physics* (2320 times).

### Productive Authors

**Figure 4:**
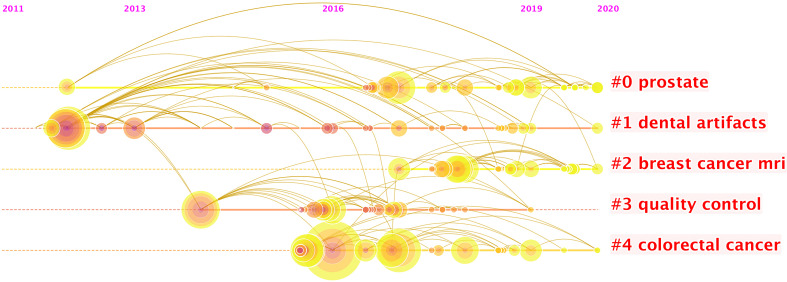
The timeline view listed authors by clustering through keywords. Each node represents one author. The position of the node here represents the time of an author’s first publication. There were 15 clusters of keywords. In each cluster, the size of each node shows the contribution of the author. It seems that the keywords “prostate” and “breast cancer MRI” occur most recently, which suggest the active participations of researchers in practicing radiomics for oncology related to them, and it also shows that researchers have been practicing radiomics research related to dental artifacts since it lasts the longest duration.

More than 2,000 authors have contributed to this field of research. **Supplementary Table S3** shows the 10 most prolific authors, and **Figure 4** presents a timeline of authors’ contributions to this field. Jie Tian is identified as the most productive author with 121 publications, and Anant Madabushi (56 publications) ranks second, followed by Dong Di (52 publications), Philippe Lambin (49 publications), and Zhenyu Liu (45 publications).

**Supplementary Table S3** shows the leading 10 authors in terms of numbers of citations. The author with the most citations is Philippe Lambin (1,171 times), followed by Alex Krizhevsky (1,090 times), Robert J. Gillies (1,066 times), Hugo J. W. L Aerts (1,052 times), and Yann Lecun (1,020 times). **Supplementary Figure S5** presents the authors’ potential cooperative relationships, as links between nodes indicate instances where authors are cited together. Since the authors on the left mainly focus on applications of radiomics while authors on the right have most laid the foundations of this field, Robert M. Haralick, shown in the middle of the network, has contributed in connecting applications and algorithms of radiomics in oncology (12).

With more than 2,000 author contributions, the knowledge map of cited authors provides information regarding the most influential authors and the collaborative relationships among them.

### Popular References

**Figure 5:**
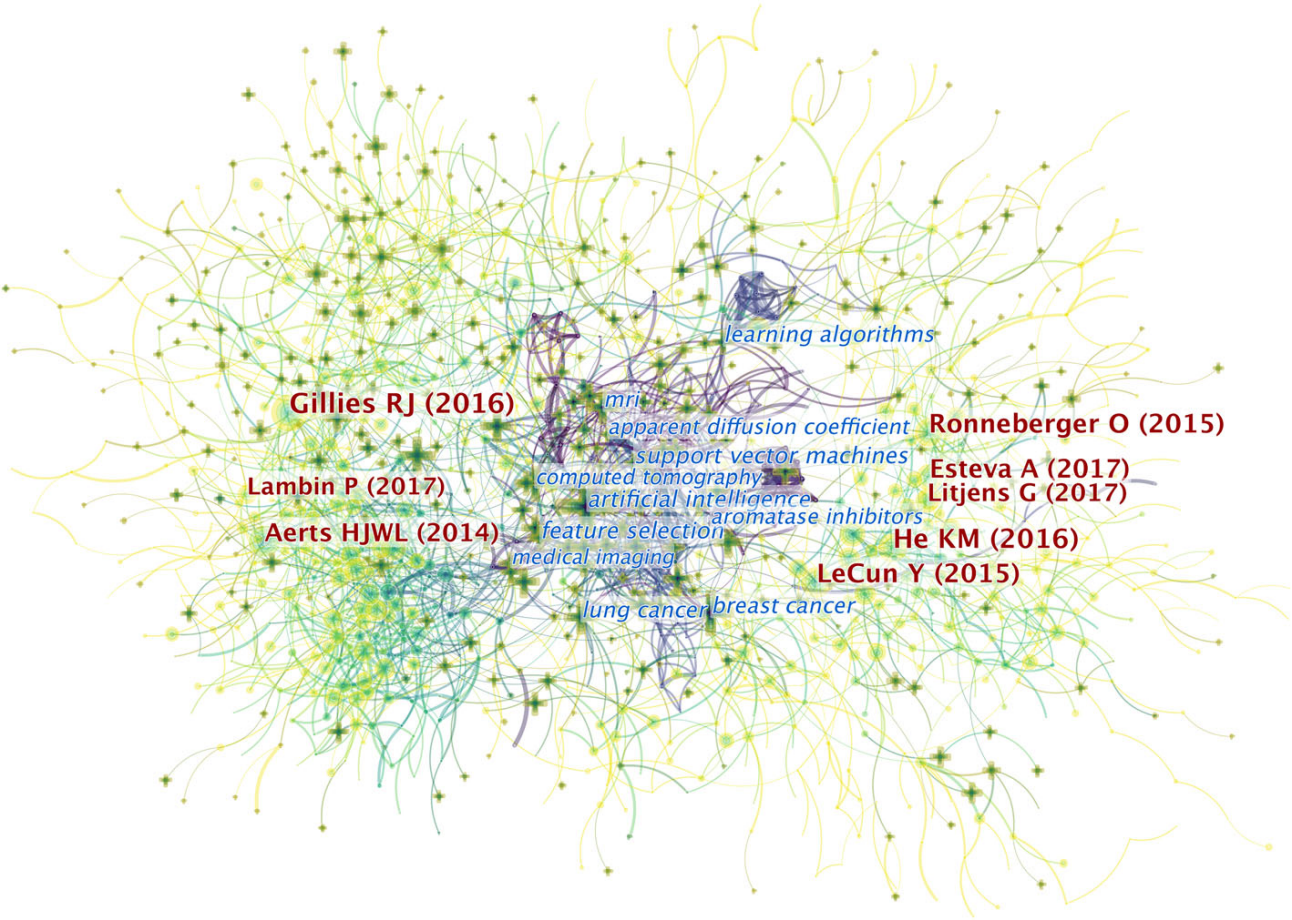
The mixed science map consists of the most cited noun phrases in publications and co-cited references in this field. By doing so, we illustrate the most co-cited references and the noun phrases in this field and uncover the relationship between them. There are two types of shapes in this picture. Each cross symbolizes a noun phrase, and each node represents a piece of co-cited references. There are links between the crosses and the circles. The links between two circles or noun phrases indicate there are some relationships between two pieces of articles or two phrases since they can be cited together. Also, the links between circles and crosses indicate that a piece of paper can be cited with certain noun phrases. Generally, there are three domains of this map. On the right and the left sides lay the most co-cited articles in the field. The ones on the left are mainly related to the definition and application of radiomics while the references on the right are mostly related to the AI algorithms involved in this field. In the middle are the most-cited noun phrases in the articles in this field. As indicated in the picture, the publications that contain these phrases are the bridges to relate publications from both sides. It illustrated that the most-cited articles from both sides may focus on conducting researches related to the noun phrases in the middle.

CiteSpace provides a mixed map of terms and co-cited references, as shown in **Figure 5**. We present the 10 most cited references in **Table 1**. Of the 6,218 publications shown, an article published by Robert J. Gillies et al. in 2016 ranks first with 1,036 citations (9). This report describes the processes, challenges, and opportunities of radiomics in detail, particularly in reference to the field of oncology.

**Table 1:** The top 10 co-cited references.

Rank	Title	Author	NOC
1	Radiomics: Images Are More than Pictures, They Are Data	Robert J. Gillies et al.	1,036
2	Deep Residual Learning for Image Recognition	Kaiming He et al.	831
3	Deep Learning	Yann LeCun et al.	741
4	U-Net: Convolutional Networks for Biomedical Image Segmentation	Olaf Ronneberger et al.	707
5	Decoding Tumour Phenotype by Noninvasive Imaging Using a Quantitative Radiomics Approach	Hugo J.W.L.Aerts et al.	638
6	Dermatologist-Level Classification of Skin Cancer With Deep Neural Networks	Andre Esteva et al.	583
7	A Survey on Deep Learning in Medical Image Analysis	Geert Litjens et al.	470
8	Radiomics: The Bridge between Medical Imaging and Personalized Medicine	Phillipe Lambin et al.	429
9	ImageNet Classification With Deep Convolutional Neural Networks	Alex Krizhevsky et al.	386
10	Fully Convolutional Networks for Semantic Segmentation	Jonathan Long et al.	377

NOC, number of citations.

**Figure 6:**
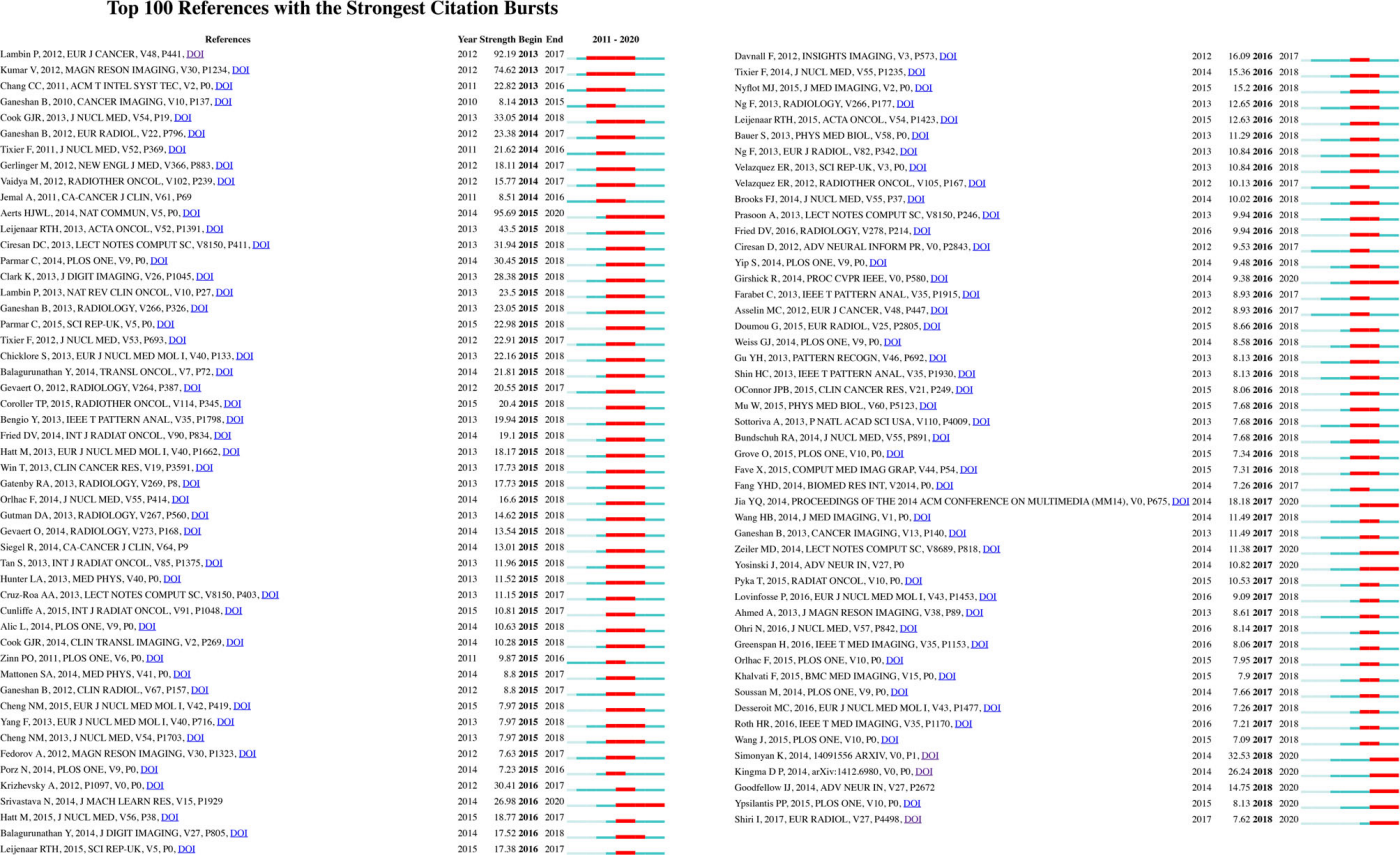
Detection of top 100 references with the strongest citation bursts.

The burst detection results show articles that have attracted the attention of peer scientists. Citation bursts note the duration and strength of each burst, or the duration and intensity of burst status, respectively (34). **Figure 6** shows the 100 references with the strongest citation bursts. The citation burst analysis shows that a publication by Hugo J. W. L Aerts et al. earned the highest burst value (95.69) from 2015 to 2020 (35). This result indicates that this publication underwent a citation burst of the highest intensity from 2015 to 2020. The article reports that the radiomics data of cancer patients contain prognostic information and are associated with underlying gene expression patterns.

### Keyword Research

**Figure 7:**
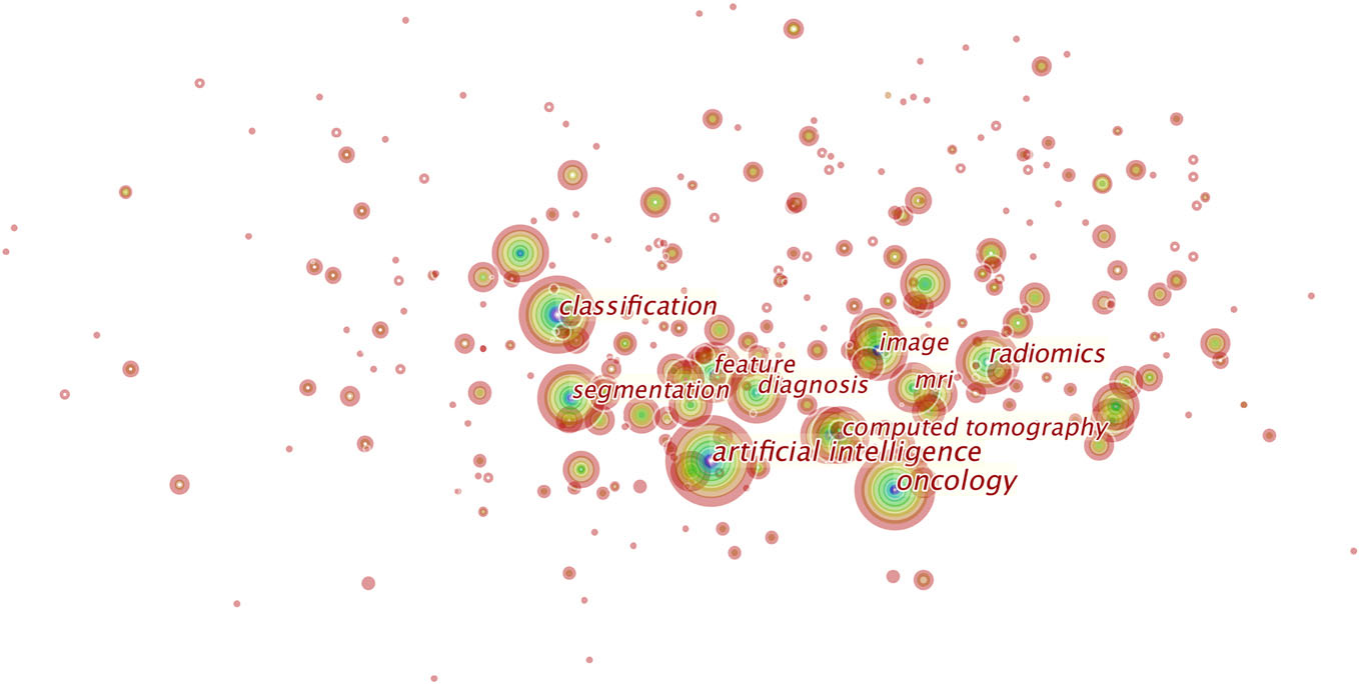
This is the knowledge map of the most cited keywords in this field. Each node represents a keyword, and the sizes of rings on the node denote the number of publications related to the keyword in a certain year. This map suggests the hotspots in this research field.

Over 800 keywords were extracted from publications. **Figure 7** shows the keywords mentioned most frequently in publications. In terms of frequency, the term “artificial intelligence” ranks first (2,931 times), followed by “oncology” (1,972 times), “radiomics” (1,437 times), “classification” (1,436 times), “diagnosis” (1,020 times), and “segmentation” (995 times).

**Figure 8:**
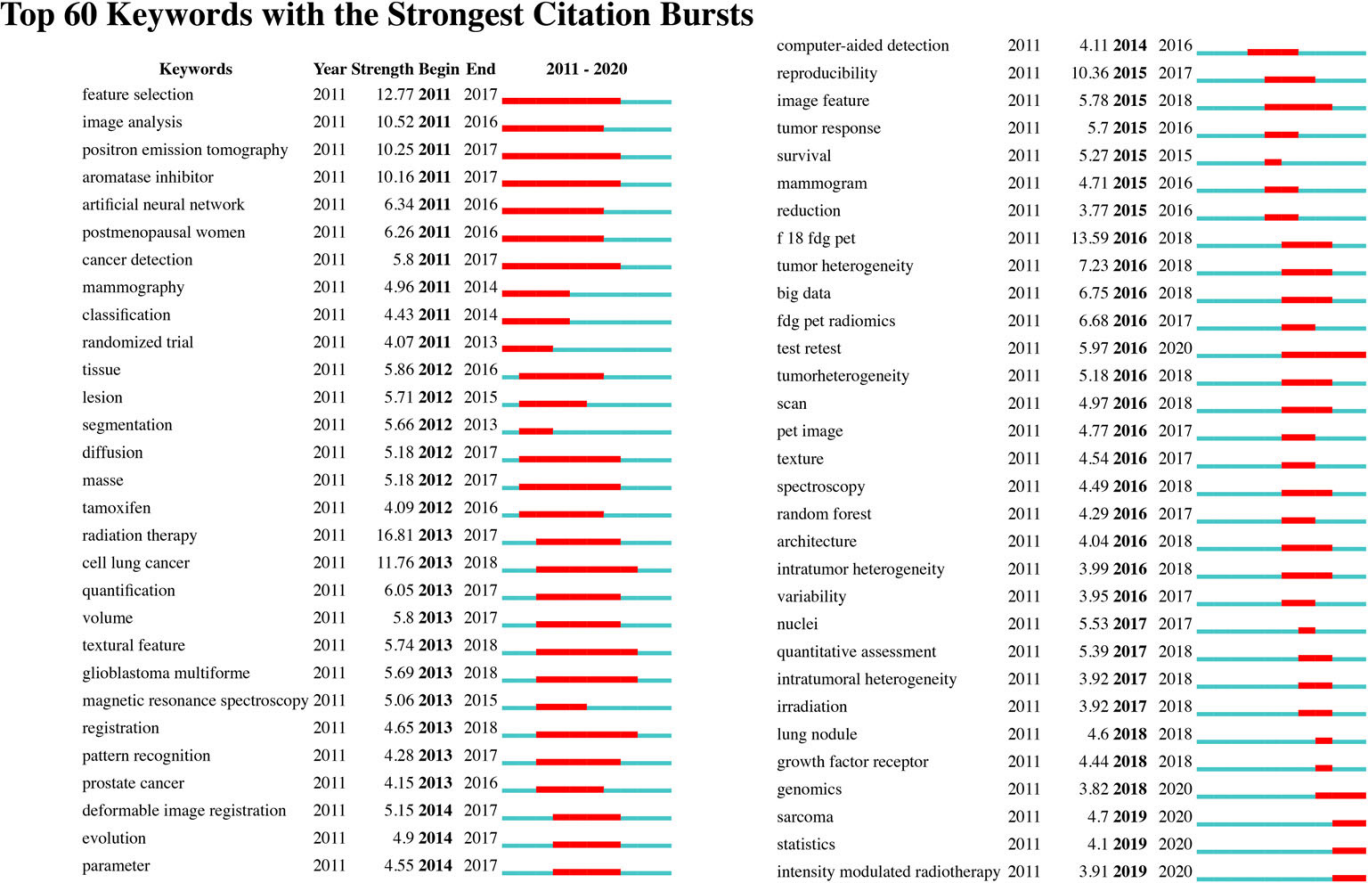
Detection of top 60 keywords with the strongest citation bursts.

We identify the top 60 keywords with citation bursts, as shown in **Figure 8**, and we provide five keywords with the strongest recent citation bursts in **Table 2**. Of the 60 keywords with the strongest citation bursts, “radiation therapy” shows the highest burst strength level of 16.81. “Test retest”, “sarcoma”, “statistics”, “intensity-modulated radiotherapy,” and “genomics” are keywords with recent citation bursts, and the term “test retest” achieves the highest burst strength and the longest duration for 2016 to 2020. These keywords indicate that the reproducibility and statistical methods of radiomics, the relationship between radiomics and genomic types, and applications of radiomics to sarcomas and intensity-modulated radiotherapy are major focuses of research in this field.

**Table 2:** Top 5 keywords in the network burst recently.

Rank	Keywords	Strength	Begin	End
1	Test retest	5.97	2016	2020
2	Genomics	3.82	2018	2020
3	Sarcoma	4.7	2019	2020
4	Statistics	4.1	2019	2020
5	Intensity-modulated radiotherapy	3.91	2019	2020

## Discussion

To our knowledge, this is the first bibliometric analysis of radiomics in oncology. This article provides an in-depth and visualized analysis of publications of this field, which may help researchers gain a basic understanding, develop areas of focus and trends and pursue further practice in this field.

The 10 leading countries include four Asian countries, two American countries, and four European countries. The USA has contributed a great volume of publications (2,280) and has collaborated frequently with other countries. Publications from the USA and China comprise >40% of all publications. Among the 10 leading institutional contributors, the Chinese Academy of Sciences ranks highest in terms of volume. Furthermore, six of the 10 leading institutional contributors are Chinese universities. However, even with the country’s large volume of publications and despite including three of the five most prolific institutions, collaboration in China has been rare and limited, suggesting that although China has been carrying out radiomics research in oncology for the last decade, extensive collaborative work is needed.

*Scientific Reports* has been the leading contributions in this field with an IF of 3.998. *Radiology* has published fewer publications but is the most cited and hence may be viewed as an influential journal in this field with a high IF of 7.931; several ground-breaking articles have been published in *Radiology* (8, 9, 35).

We list the 10 leading contributing authors and the 10 most frequently cited authors. These authors have devoted themselves to conducting research in oncology radiomics and laid the foundation of basic knowledge in this field. **Supplementary Figure S5** shows the corelationships of authors, which may indicate collaboration. With more than 100 publications, Jie Tian is the most prolific author with an H-index of 65. In 2016, to assist researchers in evaluating malignancy uncertainty, Jie Tian and his team developed a multicrop convolutional neural network to effectively characterize nodules instead of carefully segmenting using imaging and time-consuming feature extraction procedures (36). With the most citations of the 10 leading co-cited authors, Philippe Lambin is considered a pioneering and influential researcher in this field. He was the first to define radiomics and has made substantial efforts to standardize radiomics research (2, 8).

Among the 10 most cited references, an article by Robert J. Gilles et al. has been cited most, as this work provides basic information for researchers seeking to participate in radiomics research on work procedures, applications, challenges, and potential uses (9). In addition, three of the five most influential references focus on algorithms or basic knowledge of this field (37–39).

Keywords can represent areas of focus in a given field as shown in **Figure 7**, and we identified “artificial intelligence,” “tumors,” “classification,” “segmentation,” and “diagnosis” as areas of focus in this field. We summarize these areas as follows:

1. **AI**: Much of the development of radiomics relies on AI algorithms, as they can mimic human performance. To date, AI algorithms have been most widely used in radiomics in oncology. In fact, the development of AI can directly influence this field. In the past, radiomics studies most rely on traditional machine learning methods: the random forest, decision tree, and regression algorithms. These algorithms mostly rely on manual segmentation, require a huge amount of matrix manipulations, and can only perform well in small given datasets. These time-consuming processes hindered the applications of radiomics studies. Unlike the traditional machine learning methods, deep learning is a subset of AI that can acquire discriminative features from data. Instead of requiring experienced radiologists to evaluate medical images, deep learning algorithms excel at delineating and monitoring cancerous lesions and can translate medical images into quantitative data to be automatically analyzed. This approach has been most frequently applied in radiomics in oncology (40, 41). Moreover, radiomic models integrated by AI algorithms and clinical features can increase the capacity to judge individual treatment. With efforts made to develop AI technology, ground-breaking AI algorithms may enable computers to act more similar to human beings in the future.
2. **Oncology**, **radiomics**, **diagnosis**, **and classification**: When analyzing a medical image, clinicians usually depend on their personal experience, which is subjective, and results therefore vary among different radiologists (41). By applying quantitative data extracted from medical images for analysis, radiomics provides a new objective means using AI algorithms to detect lesions. Radiomics research today mainly focuses on diagnosis and classification (42, 43). For example, a computer-assisted diagnosis system can automatedly identify cancerous lesions with images and videos (44), and researchers have made considerable efforts to build radiomics models to classify identical cancerous lesions and lymph node status (5, 45).
3. **Segmentation**: To achieve reliable radiomic models in oncology, robustly and precisely delineating lesions is essential. However, traditional manual segmentation usually takes a very long time to perform, generates interobserver variability, and requires the involvement of experienced radiologists for analysis. To address these problems, automated segmentation methods have been established in radiomic research in oncology (46, 47).

Our burst detection results of references reveal articles that have attracted the attention of peer scientists (34). It seems that the ground-breaking articles and reviews with the highest citation burst strength and longest durations were published in 2015 (35). Since citation bursts may help researchers obtain a quick review of research focuses and perspectives, below we list some recent radiomic studies identified by citation burst detection that may be defined as ground-breaking works in this field in leading prospective research:

1. Some radiomic signatures have prognostic power, and there is a prognostic radiomic signature associated with underlying gene expression patterns (35);
2. Researchers have built a prostate cancer MRI computer-aided detection system evaluated on a per-patient basis and compared with the prospective performance of radiologists (48);
3. Multiparametric MRI has been used to accurately locate and segment rectal cancer (49);
4. Researchers have found that the preprocessing of CT images may influence feature volume dependence and its significance in univariate analysis models (50);
5. Using simple linear regression, a subset of radiomic features extracted from CT and cone-beam CT images can be interchangeable, and cone-beam CT radiomics can be used as a prognostic imaging biomarker (51).

We also found that radiomics research in oncology may focus on the following five keywords: test-retest, sarcoma, statistics, intensity-modulated radiotherapy, and genomics. These terms may reflect prospective areas of focus in radiomics for oncology and are summarized as follows:

1. **Test-retest and statistics**: Test-retest methods involve repeating the process of acquiring medical images to test the stability of radiomic features that they may generate. As there are many radiomics models based on various imaging parameter settings and algorithms, test-retest studies of these radiomics features have become essential for future applications. Researchers in this field have engaged in examining the reproducibility of radiomic features (52–54). However, according to a systematic review by Alberto Traverso et al., under different settings, there is no consensus on the most reproducible features (55). Moreover, the statistical method used in radiomics is of great significance. To conduct radiomic research of high quality, standardized statistical methods are essential (56, 57). Likewise, different facilities or radiologists involved during the acquisition of medical images and various analysis algorithms used may lead to bias in radiomics models. Sometimes, owing to the nonstandardized procedure of medical imaging, pictures are too distorted or of low resolution to be read by an AI algorithm. Inexperienced doctors may also fail to identify lesions. Such bias may lead to the conversion of the output result when establishing a radiomic model. Thus, future research may focus on the reproducibility of radiomics models.
2. **Sarcoma**: To date, radiomics has been used in the classification (58) and prediction of metastasis for sarcomas (59); additionally, nomograms have been used in survival prediction (60) for sarcomas. There have been relatively few radiomic analyses of sarcomas, since the prevalence of this disease is relatively low. However, this result implies that more radiomic studies should focus on sarcomas.
3. **Intensity-modulated radiotherapy**: Researchers have used radiomic models to predict patients’ responses to intensity-modulated radiotherapy (25, 61). As datasets expand, further applications of radiomics in predicting prognosis for patients undergoing radiotherapy may be identified.
4. **Genomics**: Radiogenomics involves the use of data generated by radiomic analysis to correlate with genomic patterns. Evidence has shown that radiomics features are correlated with gene patterns (35, 62, 63). Radiomic features that do not relate to gene types may supply independent information, which may enable precision medicine (9).

Our study has some limitations. First, we only focus on literature included in the WoSCC; thus, not all publications are considered and citation counts may be underestimated. Second, CiteSpace only analyses the main conclusions of publications instead of reviewing full texts; thus, some information may have been overlooked. Finally, our results only reflect the current state of radiomics research in oncology, as data are typically prone to frequent changes.

To our knowledge, this is the first analysis of radiomics in oncology conducted from the perspective of bibliometrics. The presented results may help researchers gain a basic understanding and detect areas of focus and trends and may encourage further practice in this field.

## Conclusion

Due to advances in technology, radiomics in oncology has significantly evolved over the past decade. According to results found through CiteSpace, we can conclude that current studies in this field focus on AI algorithms and on using radiomics to realize automated segmentation and classify and diagnose lesions.

Such a trend may be attributed to the rapid growth of AI algorithms, which can identify medical images. Researchers have used this new tool to train machines to identify lesions. Many articles on such issues have emerged in the last decade. This may explain why in recent decades the number of papers in this field has sharply increased. However, even with AI algorithms surpassing the performance of physicians, we must carefully validate them.

Since CNN is the most widely used AI algorithm in this field, the terms “test-retest” and “statistics” were identified during our burst detection of keywords. This finding is attributed to the characteristics of CNN itself. Unlike other algorithms, CNN requires magnitude data to train models. When such data are available, CNN can establish a robust model with high precision for clinical decision-making. However, the accessibility of medical data is always limited, and many studies mainly establish their models based on relatively small datasets. Such models may perform well only for the studied datasets. Meanwhile, a given CNN network can only perform a single defined task based on the given labels and dataset. In regard to combining several radiomic models in identifying the same oncolesions, relabeling images and retraining models may be needed due to the limitations of CNN. Therefore, model test-retest and statistical methods are likely to be widely used in future work in this field. The identification of robust features and use of standardized statistical methods may offer opportunities for the combination of various CNN models. If this can be eventually achieved, robust CNN models may be developed and may surpass the capacities of human beings. Thus, with the application of CNN models, researchers can realize their limitations. The field will thus develop with the use of a new kind of algorithm that may overcome the limitations of the CNN network (1, 64).

From our findings, we must note that not all kinds of lesions have received equal attention. According to the provided evidence, lung, breast, and prostate cancer have been the most frequently studied malignancies. According to 2020 cancer statistics (65), some of the most prevalent cancers have not been widely studied, including colon and rectum tumors, bladder cancer, kidney cancer, etc. Additionally, some region-related cancers, including liver, gastric, and oral cancer (66), which are major forms of malignancy in Asia, have been less widely reported on. This may relate to barriers of radiomics technology and poor cross-country collaboration. “Sarcoma” was highlighted in the burst detection analysis, indicating that research has focused on this field; however, only a few studies have specifically investigated this type of malignancy. This trend may be due to the relatively low disease incidence and fast progression of the disease, rendering the availability of imaging datasets more constrained. An increased use of radiomics to diagnose the above less-reported malignancies may accompany the development of AI algorithms and the sharing of databases across regions and countries.

Intensity-modulated radiotherapy and genomics may be more heavily integrated with radiomics in the future, potentially because it usually takes a long time to establish a radiomic model that can predict the survival rate of intensity-modulated radiotherapy. With the establishment of a large dataset, the use of radiomics to predict the prognoses of patients undergoing such therapy may increase in the future. Additionally, studies have found relationships between cancerous lesions and their gene patterns. Since radiomics is a risk-free means to examine the gene patterns of oncology, it may become the next area of focus of this field.

In conclusion, with active participation and regulated practices, radiomics may be applied in every phase of oncology treatment, which could further advance the development of oncology and will likely change the state of oncology imaging.

## Data Availability Statement

The original contributions presented in the study are included in the article/**Supplementary Material**. Further inquiries can be directed to the corresponding author.

## Author Contributions

YL conceived and designed the structure of this manuscript. HD, CW, NL, WS, QZ, YH, and ZJ wrote the paper. YL revised the paper. All authors contributed to the article and approved the submitted version.

## Funding

This work was supported by the National Natural Science Foundation of China [Grant No. 81972546 to YL].

## Conflict of Interest

The authors declare that the research was conducted in the absence of any commercial or financial relationships that could be construed as a potential conflict of interest.

## Publisher’s Note

All claims expressed in this article are solely those of the authors and do not necessarily represent those of their affiliated organizations, or those of the publisher, the editors and the reviewers. Any product that may be evaluated in this article, or claim that may be made by its manufacturer, is not guaranteed or endorsed by the publisher.
