# Supplementary material for: Radiomics in Oncology: A 10-Year Bibliometric Analysis
Source: Front Oncol. 2021 Sep 20;11:689802. doi: 10.3389/fonc.2021.689802 (PMC8488302; doi:10.3389/fonc.2021.689802)
Supplement: Supplementary file 1 [file DataSheet_1.pdf]

Supplementary Material

1 Supplementary Figures

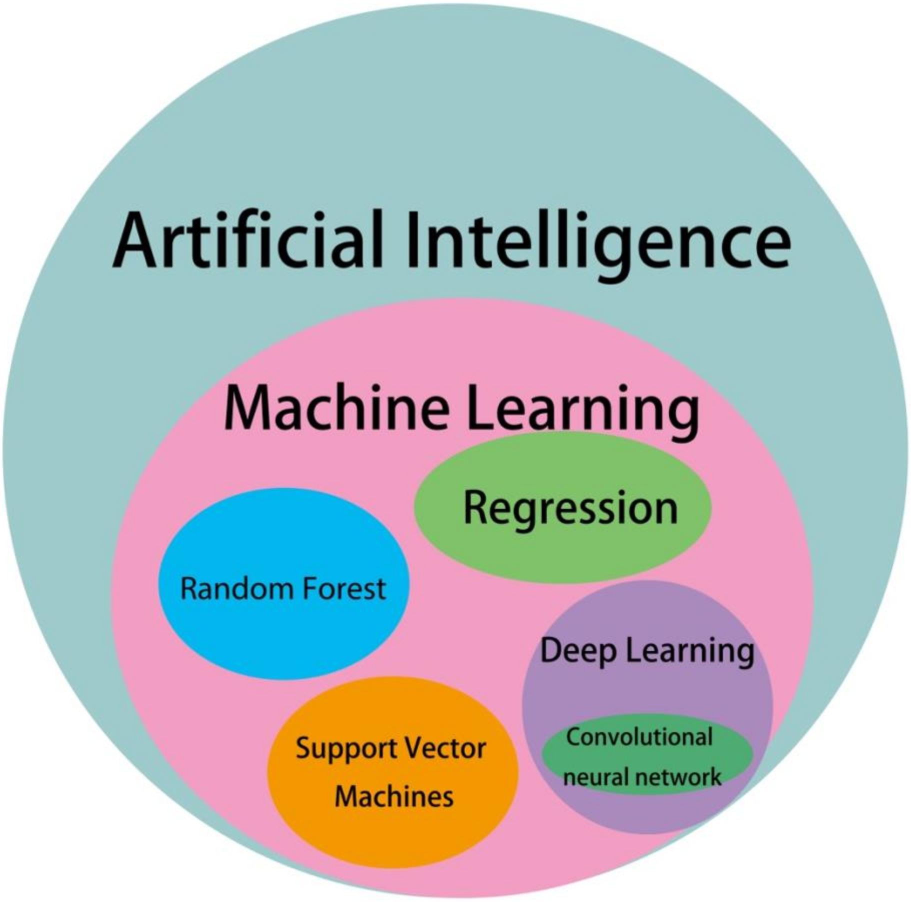

Supplementary Figure 1. The relationship between the algorithms.

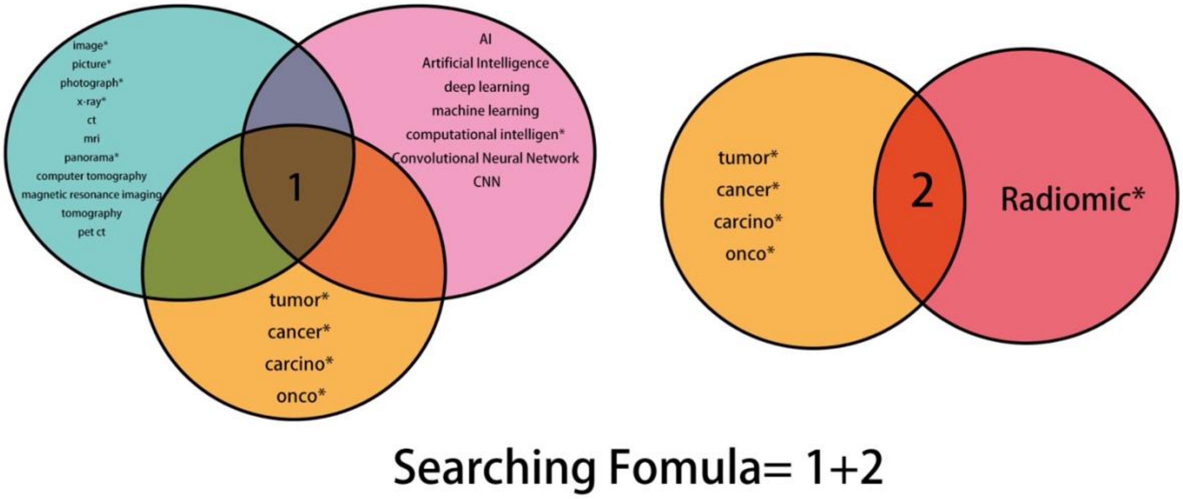

Supplementary Figure 2. The searching formula of this article.

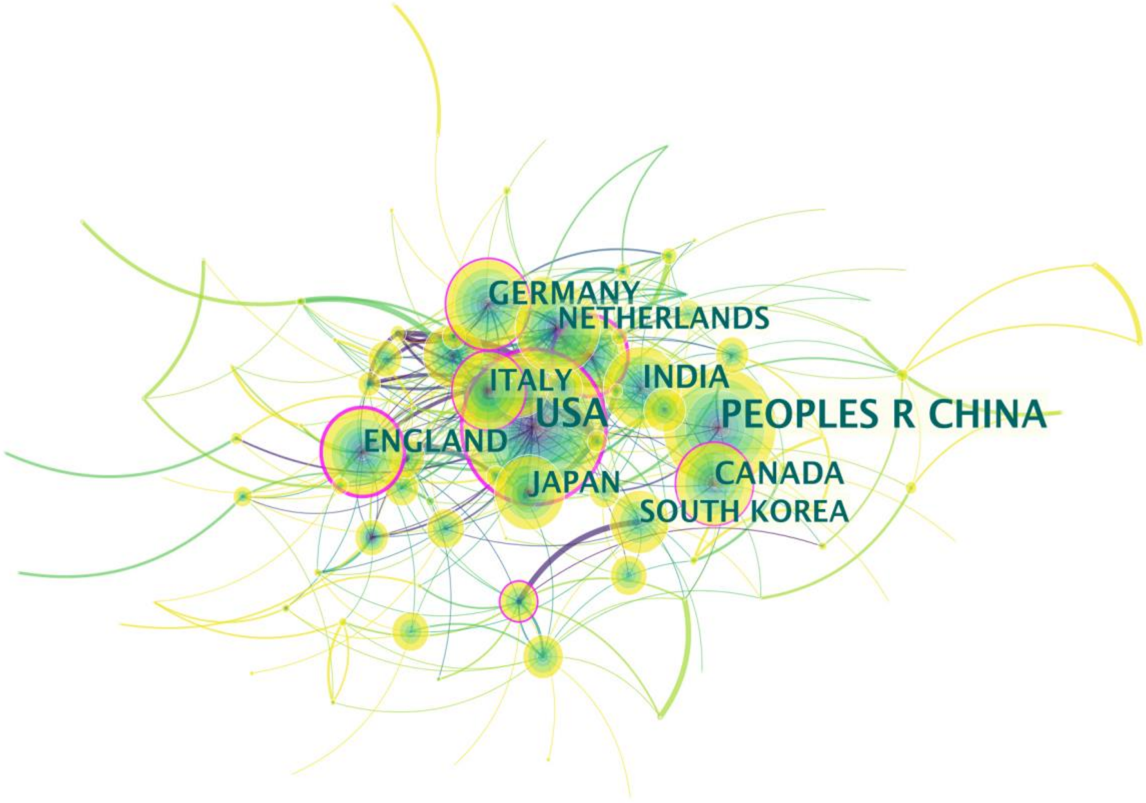

Supplementary Figure 3. The knowledge map of active countries practicing radiomics research for oncology from 2011 to 2020. Each node represents one country, and different colors of the arced lines represent different years. The thickness of the arced line means the different number of publications in each year. Links between two nodes indicate that there was a collaboration between the two countries. Colors of links represent different years of the first collaboration, and the width of links denote the strengths of their collaborations. The size of rings on the nodes indicates the number of publications for each year. The purple rings out of the circles indicate the high centrality of each node.

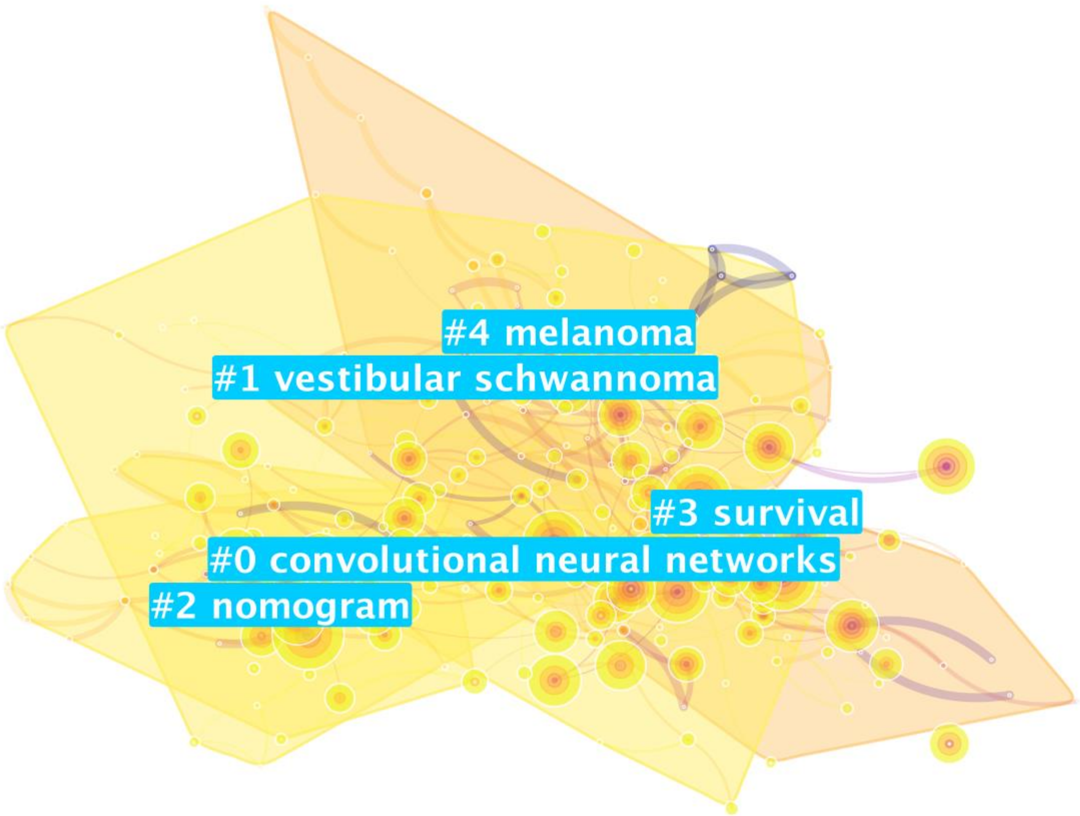

Supplementary Figure 4. The knowledge map of institutions participating in practicing radiomics for oncology. Each node represents one institute, and the color of the circles of each node represents different years, the thickness of them stands for the number of publications in certain years. Lines between circles symbolize the collaboration between two institutions, different colors, and sizes of different lines represent different years and the strength of the collaborations. By clustering, the keywords of institutions were divided into 14 clusters among the institutions, and we listed the largest five of them. Institutions in the same cluster collaborate more frequently, and the labels represent the keywords of clusters, for example, institutions in cluster #0 may focus mainly on convolutional neural networks.

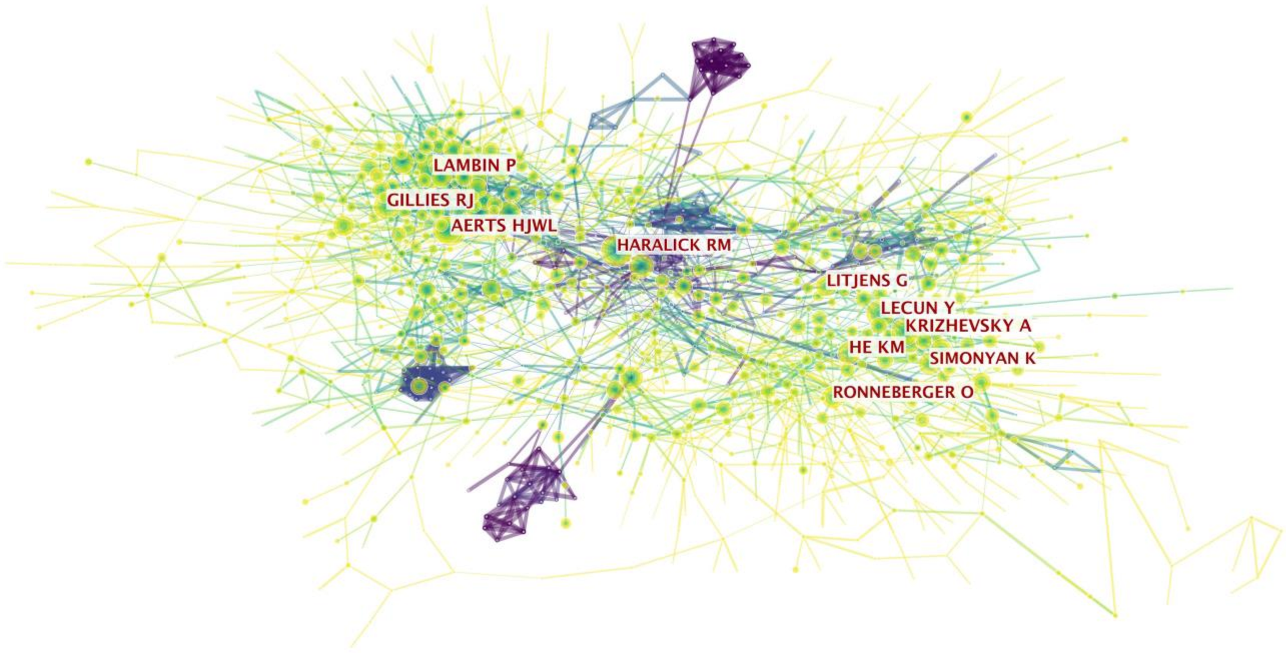

Supplementary Figure 5. The knowledge map presents the co-cited authors of this field. Each circle represents one co-cited author. The thickness of the arced lines on the circles indicates the times of the authors being co-cited. The line occurs when two authors were cited together, which may indicate further potential cooperation among the authors. We listed the top 10 most co-cited authors here. The authors on the left mostly practice radiomics in oncology, while the authors on the right were dedicated to improving the base of applications for radiomics in oncology. And Robert M. Haralick occurs in the middle domain of this map, which indicates this author can be co-cited with both sides of the authors.

2. Supplementary Tables

Supplementary Table 1. Top 10 prolific countries and institution

Rank	Country/ Institution	NOP
	**Top 10 prolific country**	
1	The United States	2280
2	China	2136
3	India	456
4	Germany	454
5	England	401
6	Canada	368
7	Italy	364
8	South Korea	362
9	Japan	335
10	Netherlands	321
	**Top 10 prolific institution**	
1	Chinese Academy of Sciences (CN)	224
2	Sun Yat-Sen University (CN)	168
3	Fudan University (CN)	155
4	Harvard Medical School (US)	145

5	Stanford University (US)	136
6	University of Chinese Academy of Sciences (CN)	129
7	The University of Texas MD Anderson Cancer Center (US)	128
8	Memorial Sloan-Kettering Cancer Center (US)	126
9	Shanghai Jiao Tong University (CN)	123
10	Beihang University (CN)	119

Note: NOP, number of publications; CN, China; US, United States

Supplementary Table 2. Top 10 prolific/co-cited journals

Rank	Journal name	NOP	IF*
	**Top 10 prolific journal**		
1	Scientific Reports	253	3.998
2	Medical Physics	244	3.317
3	IEEE Access	241	6.685
4	European Radiology	213	4.101
5	Frontiers in Oncology	180	4.898
6	Physics in Medicine and Biology	143	2.883
7	Plos One	122	2.74
8	IEEE Transactions on Medical Imaging	117	6.685
9	Journal of Magnetic Resonance Imaging	103	3.954
10	Journal of Medical Imaging	97	N/A
	**Top 10 co-cited journal**		
1	Radiology	3258	7.931
2	IEEE Transactions on Medical Imaging	2800	6.685
3	Scientific Reports	2410	3.998
4	Plos One	2407	2.74
5	Medical Physics	2320	3.317

6	Lecture Notes in Computer Science	2207	N/A
7	Medical Image Analysis	2024	11.148
8	European Radiology	1973	4.101
9	IEEE Conference on Computer Vision and Pattern Recognition	1806	N/A
10	Nature	1793	42.779

Note: NOP, number of publications; IF: impact factor; *Calculated in 2019.

Supplementary Table 3. Top 10 prolific/cited authors

Rank	Journal name	NOP	H-Score*
	**Top 10 prolific authors**		
1	Jie Tian	121	65
2	Anant Madabushi	56	37
3	Di Dong	52	24
4	Phillipe Lambin	49	32
5	Zhenyu Liu	45	20
6	Hugo J. W. L Aerts	45	49
7	Robert J. Gillies	39	85
8	Zaiyi Liu	33	23
9	Wei Wang	32	27
10	Yuanyuan Wang	29	24
	**Top 10 cited authors**		
1	Phillipe Lambin	1171	32
2	Alex Krizhevsky	1090	6
3	Robert J.Gillies	1066	85
4	Hugo J. W. L Aerts	1052	49
5	Yann Lecun	1020	47

6	Kaiming He	984	42
7	Olaf Rnneberger	819	29
8	Karen Simonyan	803	18
9	Robert M.Haralick	705	40
10	Geert Litjens	672	27

Note: NOP, number of publications; *Calculated in recent 5-years.
